# A Student-Centered Approach Towards Implementing Large Language Models (LLMs) in Medical Education

**DOI:** 10.1007/s40670-026-02675-x

**Published:** 2026-03-21

**Authors:** Lancelot P. Herpin, Pratik S. Vadlamudi, Rukam Mahawa, Mark H.S. Troftgruben, Joanna Hua, C. Jessica Dine, Andrew Hashikawa

**Affiliations:** 1 Perelman School of Medicine, University of Pennsylvania, 3400 Civic Center Blvd, Philadelphia, PA 19104 USA; 2 University of Michigan Medical School, Ann Arbor, MI USA; 3 Trinity Health Livonia Hospital, Livonia, MI USA

**Keywords:** Artificial intelligence, Medical education, Large-language models, Curriculum development

## Abstract

**Introduction:**

Artificial intelligence (AI) generative text tools built on large language models (LLMs) are used by medical students, often without formal training. Research on students’ use of LLMs is limited. This study examined students’ self-reported experiences with LLMs and their expectations for the integration of LLMs in medical education.

**Materials and methods:**

A 30-item cross-sectional email-based survey was distributed at two US medical schools. Survey results were analyzed using descriptive statistics, with non-parametric tests, and ordinal logistic regression was used to assess patterns of LLM engagement. Respondents were stratified into low, moderate, and high-LLM usage groups using k-means clustering, and heatmaps of user frequency were generated.

**Results:**

A total of 103 students, spanning all levels of training within medical school, completed the survey. High- versus low-usage users reported greater LLM knowledge (3.46±0.82 vs 2.40±0.91, *p* = 0.0004), more confidence judging LLM HIPAA-compliance (*p* = 0.016), stronger agreement that LLMs improve medical education (4.14±0.79 vs. 2.16±1.19, *p* < 0.0001), and more LLM clinical application predictions (*p* = 0.0001). Usage (*p* = 0.007), not institution (*p* = 0.714) predicted educator-driven LLM engagement. LLMs were most helpful for fact-finding, literature summarization, and differential diagnosis and least helpful for flashcards. High-usage users noted inaccuracies more frequently (*p* <0.0001). Students anticipated future roles in documentation, administrative tasks, literature review, and patient education. Most (86.4%) endorsed formal training, emphasizing critical thinking and ethical/legal skills.

**Discussion:**

Medical students’ perceptions and confidence in LLMs are associated with usage. While students see potential in reducing administrative burden, they also express concern about impacts on learning. Respondents highlighted topics that should be included in formal LLM education.

**Supplementary Information:**

The online version contains supplementary material available at 10.1007/s40670-026-02675-x.

##  Introduction

Recent advances in artificial intelligence (AI) have accelerated its integration into the mainstream of society and expanded its range of practical applications. The use of AI in education has been studied for decades as a potential means to support personalized and adaptive learning [1, 2]. The emergence of freely accessible generative AI tools, powered by large language models (LLMs) such as ChatGPT (OpenAI, 2023), Perplexity (Perplexity AI, 2023), and Claude (Anthropic, 2023), has renewed concerns within education about plagiarism, academic integrity, and the responsible use of AI in learning [3]. 

LLMs can generate complex text and synthesize information from large datasets, capabilities that are rapidly transforming both education and healthcare [4–6]. AI has already been employed in clinical applications of healthcare, but the use and integration in education remains unexplored [7]. As these tools become more advanced and prevalent, medical education must adapt, including how students learn and work. Several digital health competency frameworks have recently been developed, including the 27 artificial intelligence-specific competencies for medical education and the Digital Health Competencies in Medical Education (DECODE) framework to guide implementation of digital health within medical education globally, with specific attention to competencies for using artificial intelligence in healthcare [8, 9]. These competencies span understanding healthcare datasets and how artificial intelligence can improve prediction, ensuring real-world validation and the safe, ethical, legally compliant use of AI, integrating outputs with clinical judgment and workflows, maintaining foundational data science/statistics knowledge, selecting fit-for-purpose tools, and collaborating across disciplines while staying current with the evidence.

While medical students nationwide are already leveraging AI tools to supplement their learning, use of LLMs often occurs without clear guidelines, training, or a comprehensive understanding of the tools’ limitations, benefits, and data privacy concerns. As LLM technology evolves, medical education programs will need to integrate structured, evidence-based approaches to guide their effective use. Prior studies on AI and LLMs in medical education have explored personalized learning, content accuracy, and examination performance [10]. Letters from medical students and educators are found in the literature focusing on the impacts on physician employability, writing of journal articles, medical examinations, and model accuracy [10–12]. However, the literature remains limited and has focused more on how educators might deploy LLMs than on how medical students use them and the associated educational outcomes [13–15]. To address this gap, this study examined students’ self-reported experiences with LLMs and their expectations for the integration of these tools into medical education, using elements from two digital health frameworks to inform our survey questions and topic domains. Our findings underscore that medical education provides insufficient training in artificial intelligence, leaving students underprepared; medical schools should respond by systematically assessing digital and LLM literacy and implementing structured curricula and institutional safeguards to support responsible use.

## Materials and Methods

### Study Design

A cross-sectional, web-based survey was administered to medical students at the Perelman School of Medicine at the University of Pennsylvania (PSOM) and the University of Michigan Medical School (UMMS). Institutional Review Board (IRB) approval was obtained from both institutions (University of Pennsylvania IRB #858189; University of Michigan IRB #HUM00267318).

The online survey was distributed via email to the entire student body at each institution using school-wide listservs, including MD-PhD and combined-degree or year-out (CDYO) students (those pursuing a master’s degree or a dedicated research year). The initial recruitment email was sent in April 2025, with one follow-up reminder sent two weeks later. The survey remained open for one month. The invitation email and the introductory section of the survey described the study purpose, emphasized confidentiality and anonymity, and explained that participation was voluntary. Consent was implied through completion and submission of the survey. To mitigate potential non-response bias, we used a brief survey format, sent an initial invitation with a reminder 2 weeks later, and offered entry into a gift card raffle; anonymity was emphasized by not collecting identifying information and using a separate, unlinked form for raffle email addresses.

### Survey Development

Survey items were developed following a comprehensive literature review on ChatGPT and AI in medical education, focusing on the 27 artificial intelligence-specific competencies and the DECODE framework [8, 9]. The survey instrument was iteratively revised by two medical educators, one of whom had expertise in bioinformatics. The final survey was implemented using *Google Forms* (Google LLC, Mountain View, California) and pilot-tested on 10 medical students to ensure question clarity and time feasibility. Minor revisions were made based on pilot feedback, and those responses were excluded from further analysis.

The final instrument consisted of 30 items organized into four sections (Appendix 1), including multiple-choice, multi-select, and free-response formats. Participants were able to modify their responses until final submission. The survey assessed students’ perceptions, experiences, and expectations regarding the use of generative LLMs in medical education and clinical practice. Basic demographic information was collected at the end of the survey. Upon completion, students were invited to enter an optional raffle for one of five $25 online shopping gift cards via a separate, unlinked form to maintain anonymity.

### Data Analysis

Data were analyzed using R (version 4.2.2; R Foundation for Statistical Computing). Descriptive statistics summarized the frequency of responses for each item. Internal consistency of the four-item self-rated large language model (LLM) knowledge scale (capabilities, applications, prompt engineering, and limitations/challenges) was assessed using Cronbach’s alpha. Participants were classified into three subgroups based on their frequency of LLM use via k-means clustering. These subgroups were then used to evaluate differences in participant responses. Statistical significance was assessed using the Kruskal-Wallis rank sum test, with Dunn’s post hoc tests conducted to identify specific pairwise differences. To analyze responses regarding faculty-prompted LLM engagement, a proportional odds ordinal logistic regression model was employed. Medical school affiliation was included as a covariate, given its potential influence on educator-driven LLM use.

## Results

Sixty-one of the 791 (7.7%) full-time students at the University of Michigan Medical School and 42 of the 801 (5.2%) at the Perelman School of Medicine completed the survey. A total of 83 participants (80.6%) across all stages of medical school (traditionally: after university, before medical residency) completed the demographics section (Table 1).

**Table 1:** Survey participant demographics*

Demographic Characteristic	Participants, *n* (%)
Gender (*n* = 83)	
Male	37 (44.6%)
Female	45 (54.2%)
Non-binary	1 (1.2%)
Race (*n* = 83)	
Caucasian	47 (56.6%)
Asian	29 (34.9%)
African American	1 (1.2%)
Middle Eastern	2 (2.4%)
Mixed	3 (3.7%)
Unknown	1 (1.2%)
Ethnicity (*n* = 83)	
Hispanic/Latino	10 (12%)
Non-Hispanic/Latino	73 (88%)
Age (*n* = 83)	
21–25	34 (41.5%)
26–30	41 (50.0%)
31–35	7 (8.5%)
Year in school (*n* = 103)	
Pre-clerkship	24 (23.3%)
Clerkship	16 (15.5%)
Post-clerkship	51 (49.5%)
CDYO	6 (5.8%)
MSTP - G1	1 (1.0%)
MSTP - G2	1 (1.0%)
MSTP - G3	2 (1.9%)
MSTP - G4	2 (1.9%)

*A total of 83 participants (80.6%) completed the optional demographic section

### Current Use of LLMs in Medical Education

**Fig. 1:**
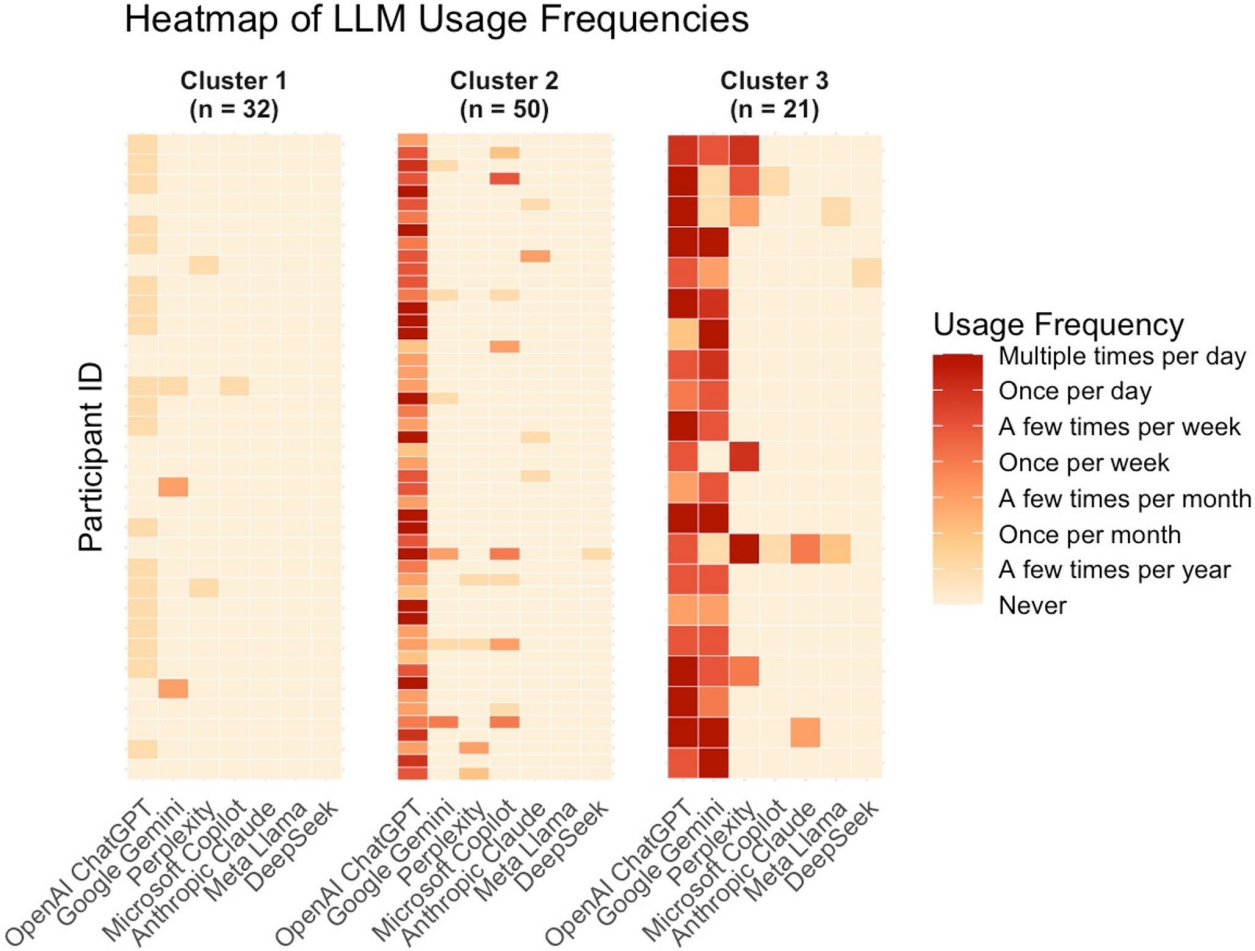
Heatmap displaying the frequency of use across various large language models (LLMs), with participants stratified into three clusters using K-means clustering: (1) Low/Non-Users, (2) Moderate/Occasional Users, and (3) High/Frequent Users

While OpenAI ChatGPT was the most frequently used LLM, multiple models were represented in the survey (Table 2). K-means clustering of reported usage frequencies identified three groups: (1) Low/Non-Users, (2) Moderate/Occasional Users, and (3) High/Frequent Users (Fig. 1). These clusters were used for stratified analysis to assess how usage frequency of participants affected their responses.

**Table 2:** Participant frequency of use of different LLMs (*n* = 103)

**LLM**	**Any Use, n (%)**	**Weekly Use, n (%)**	**Daily Use, n (%)**
OpenAI ChatGPT	90	51	26
Google Gemini	29	16	7
Perplexity	12	5	3
Microsoft Copilot	12	3	0
Anthropic Claude	6	1	0
Meta Llama	2	0	0
DeepSeek	2	0	0
OpenEvidence	21	-	-
UM-GPT	6	-	-
Other	4	-	-

**Fig. 2:**
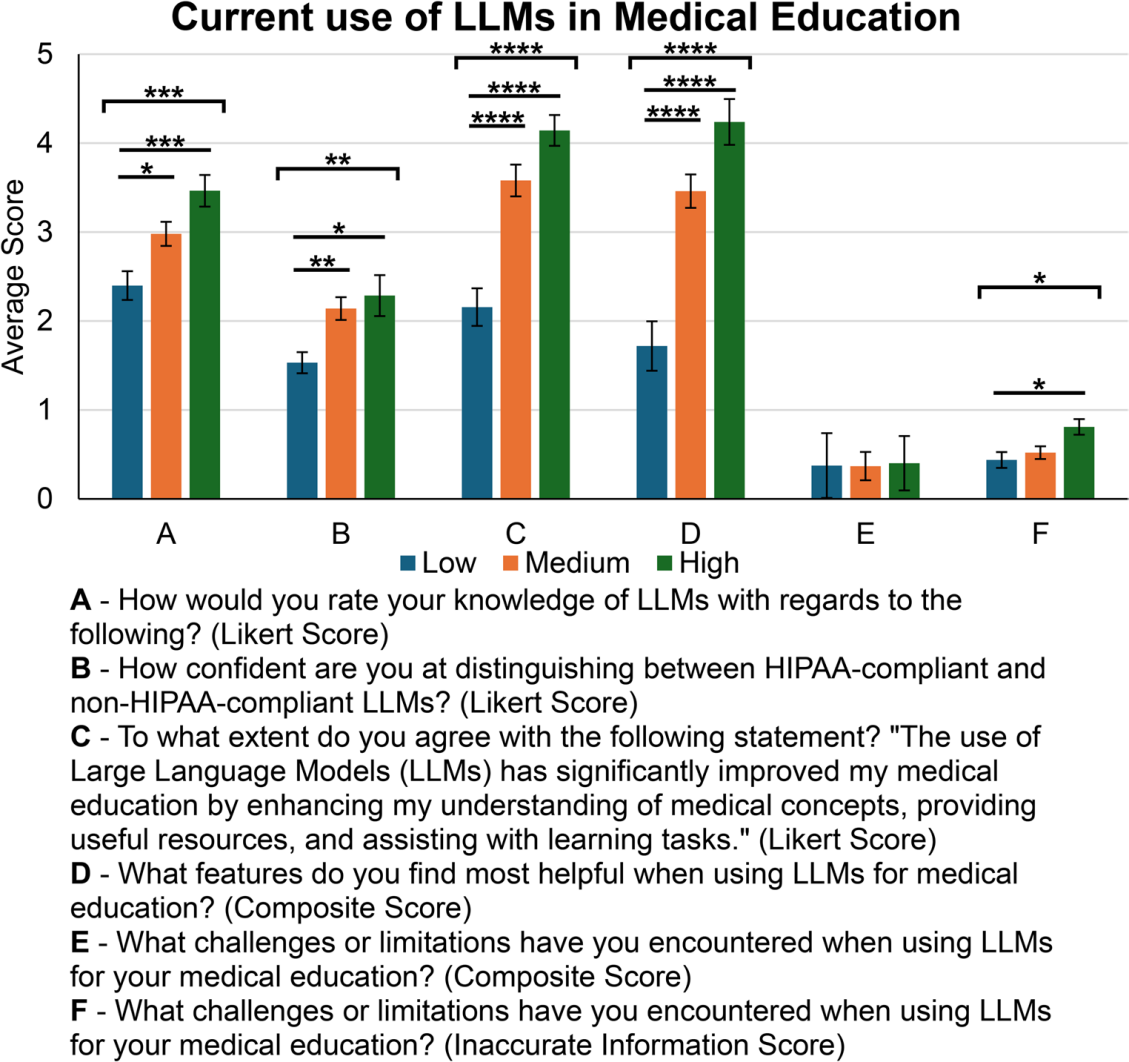
Summary of average scores and statistical tests for current use of LLMs in medical education. Kruskal-Wallis (shown with brackets) was used to test for any difference between low-, medium-, and high-LLM-use categories, with Dunn’s Post Hoc Test to test for specific comparisons (shown with line). * = *p* < 0.05; ** = *p* < 0.01; *** = *p* < 0.001; **** = *p* < 0.0001

Self-rated knowledge about LLM capabilities, applications, prompting, and limitations (on a 5-point Likert scale) was higher in high usage groups than in both moderate and low usage groups (Fig. 2A). The LLM knowledge scale demonstrated high internal consistency (Cronbach’s alpha = 0.94; average inter-item correlation = 0.81).

Seventeen (16.5%) respondents reported paying for LLM access.

**Fig. 3:**
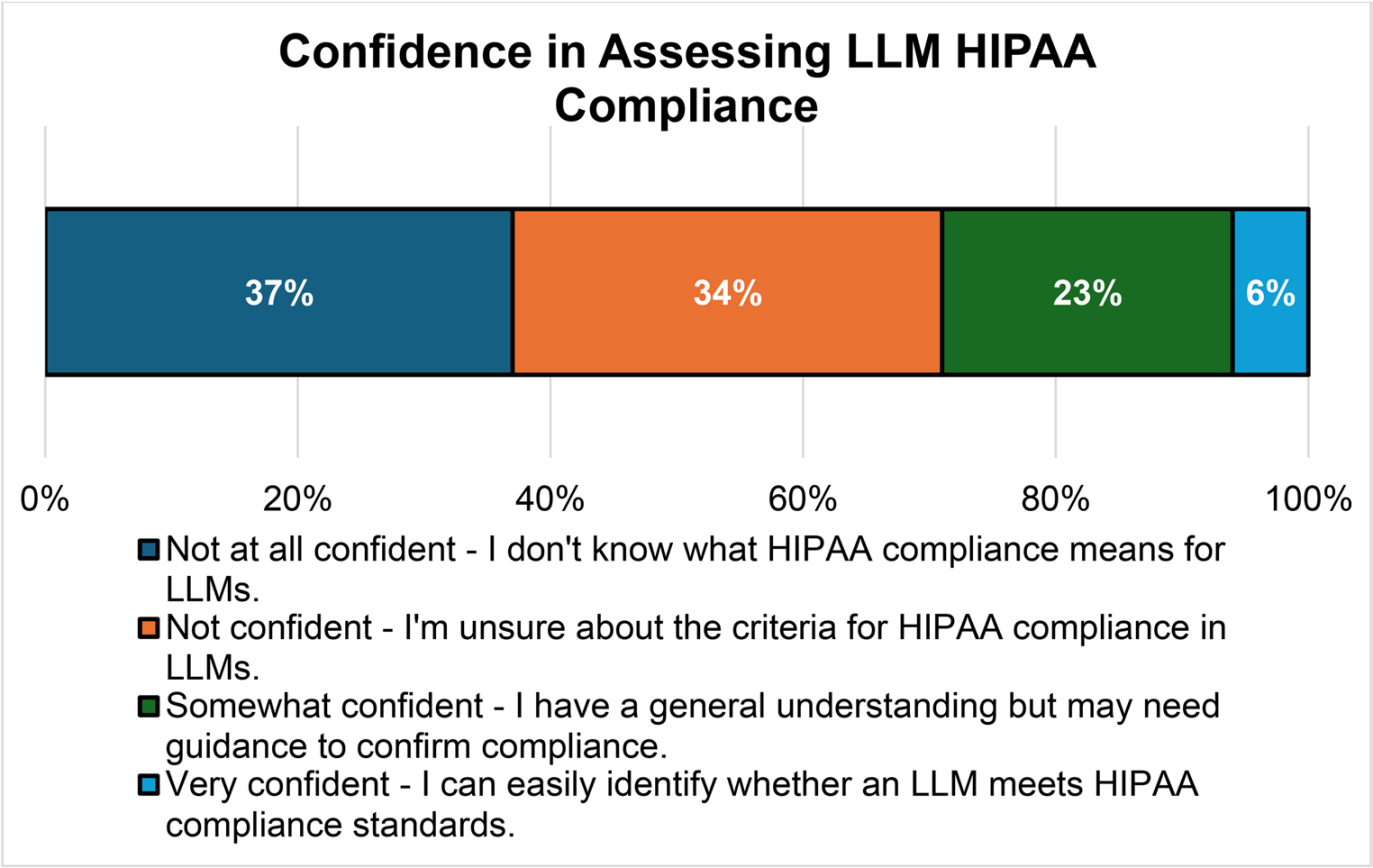
Percentage bar chart displaying respondents’ confidence in assessing LLM HIPAA compliance

Eleven (10.7%) respondents used a HIPAA-compliant LLM. Confidence in evaluating LLM HIPAA-compliance (on a 4-point Likert scale) varied by usage group (Fig. 2B), with most respondents choosing either “not at all confident” or “not confident” in their ability to assess HIPAA compliance in LLMs (Fig. 3).

In medical education, participants most commonly used LLMs to look up fact-based questions and summarize medical literature, and least commonly to generate flashcards and clinical vignettes or to summarize patient information (Table 3).

**Table 3:** Summary of responses on current use of LLMs in medical

In which of the following medical school contexts have you used LLMs, and how helpful have they been?			
	Percent Usage		Percent Helpful^*^
Looking up fact-based questions	71.8%		91.9%
Summarizing medical literature	56.3%		93.1%
Generating a differential diagnosis	52.4%		90.7%
Assisting with research data analysis	39.8%		85.4%
Assisting with writing research abstract/manuscript	39.8%		90.2%
Generating explanations to review multiple-choice questions	37.9%		84.6%
Generating practice questions	35.9%		73.0%
Generating interactive clinical vignettes	33.0%		79.4%
Summarizing clinical vignettes	24.3%		100.0%
Summarizing patient information	14.6%		86.7%
Generating flash cards	11.7%		41.7%
**What features do you find most helpful when using LLMs for medical education?**			
	**Participants**,** n (%)**		
Speed of Information Retrieval	74 (71.8%)		
Clarity of explanations	66 (64.1%)		
Ability to generate detailed responses to complex questions	65 (63.1%)		
Personalized or tailored responses to specific queries	64 (62.1%)		
Help with study organization or planning	25 (24.3%)		
Availability of interactive, case-based learning	23 (22.3%)		
Summarizing scientific papers/understanding new coding tools for research	2 (1.9%)		
**What challenges or limitations have you encountered when using LLMs for your medical education?**			
	**Participants**,** n (%)**		
Inaccurate (factually incorrect) information	57 (55.3%)		
Lack of depth in clinical reasoning	53 (51.5%)		
Limited ability to answer highly specialized questions	48 (46.6%)		
Lack of clinical context in responses	41 (39.8%)		
Ethical concerns (e.g., misinformation, privacy)	36 (35.0%)		
Outdated (not current) information	28 (27.2%)		
Difficulty understanding complex medical jargon	9 (8.7%)		
Concerns about energy usage/environmental impact	2 (1.9%)		
Not organized in helpful way/unfocused	2 (1.9%)		

*Percentages reflect helpfulness among respondents who used LLMs in each context

The most helpful LLM features were speed of information retrieval and clarity of explanations, while the least helpful features were summarizing new evidence and case-based interactive learning (Table 3), with moderate- and high-usage participants listing significantly more helpful features compared to low-usage participants (Fig. 2D).

The top reported limitations were inaccurate information and a lack of clinical reasoning depth (Table 3), with concerns about inaccurate information more noted among high-usage than low-usage respondents (Fig. 2F). However, the total number of reported limitations did not differ by usage group (Fig. 2E). In free response boxes, respondents reported identifying inaccuracies primarily by cross-checking with medical literature or faculty guidance, recognizing fabricated or outdated citations, spotting logical inconsistencies, and relying on their prior medical knowledge.

**Fig. 4:**
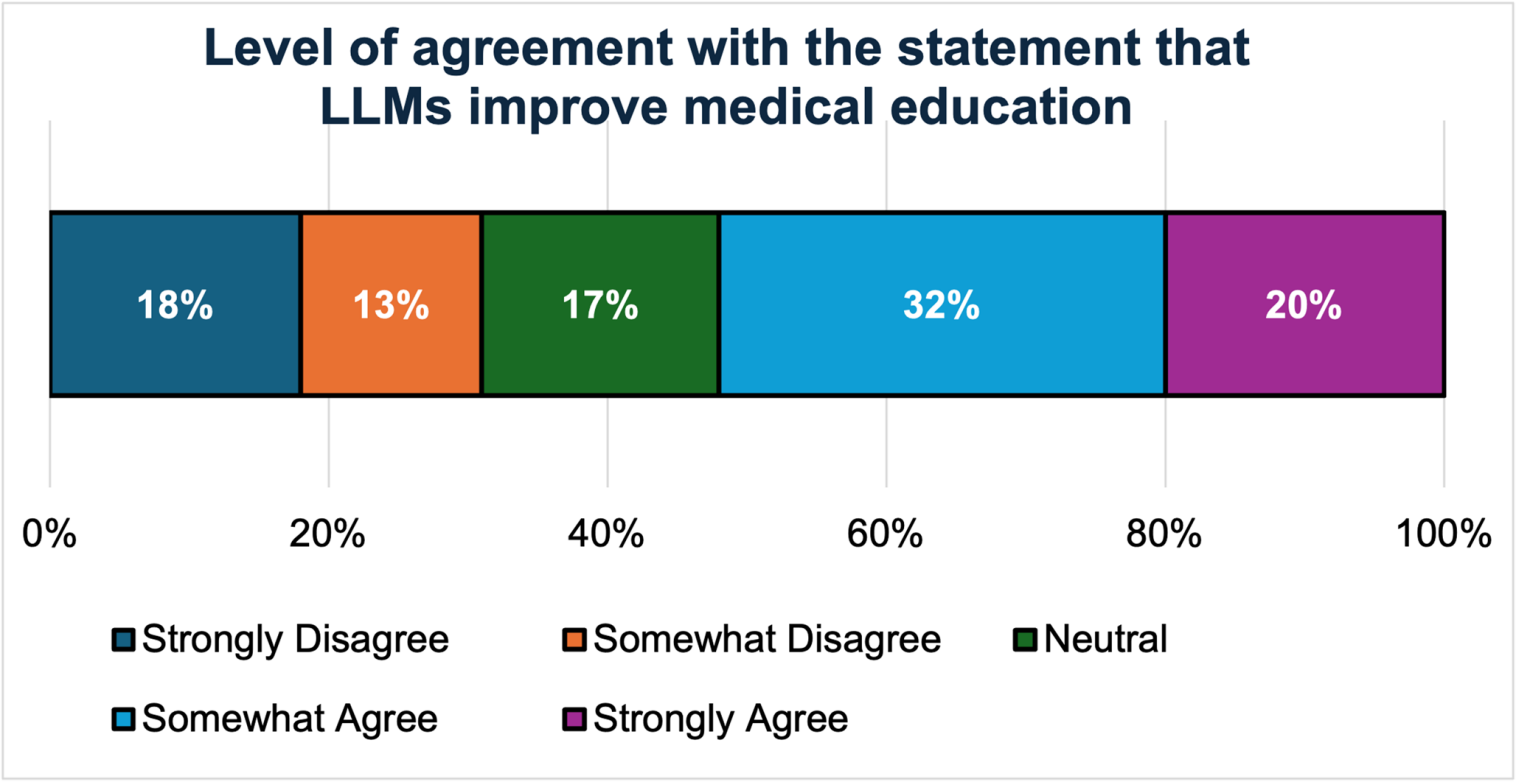
Percentage bar chart displaying respondents’ agreement with whether LLMs improved medical education

Agreement with the statement that LLMs improve medical education (on a 5-point Likert scale) also differed significantly by usage group, with high users reporting the greatest agreement, followed by moderate users, and then low users (Figs. 2C and Fig. 4).

In a proportional odds logistic regression model, institutional affiliation did not predict educator-driven LLM engagement (*p* = 0.714), but cluster membership did (*p* = 0.007), with higher-use clusters more likely to report educator-driven LLM engagement.

### Predictions for Future Integration of LLMs in Medical Practice

Free-text responses indicated that respondents expect LLMs to enhance medical education by summarizing complex topics and generating personalized study materials, such as multiple-choice questions and clinical vignettes. Respondents also predicted LLMs would support clinical tasks (e.g., generating differential diagnoses, interpreting imaging, evaluating literature for evidence-based decisions), administrative tasks (e.g., note-writing, email drafting), research (e.g., writing, presentations), and generation of patient education materials. However, many students expressed concerns that overreliance on LLMs could hinder the development of core medical knowledge and clinical reasoning skills.

**Fig. 5:**
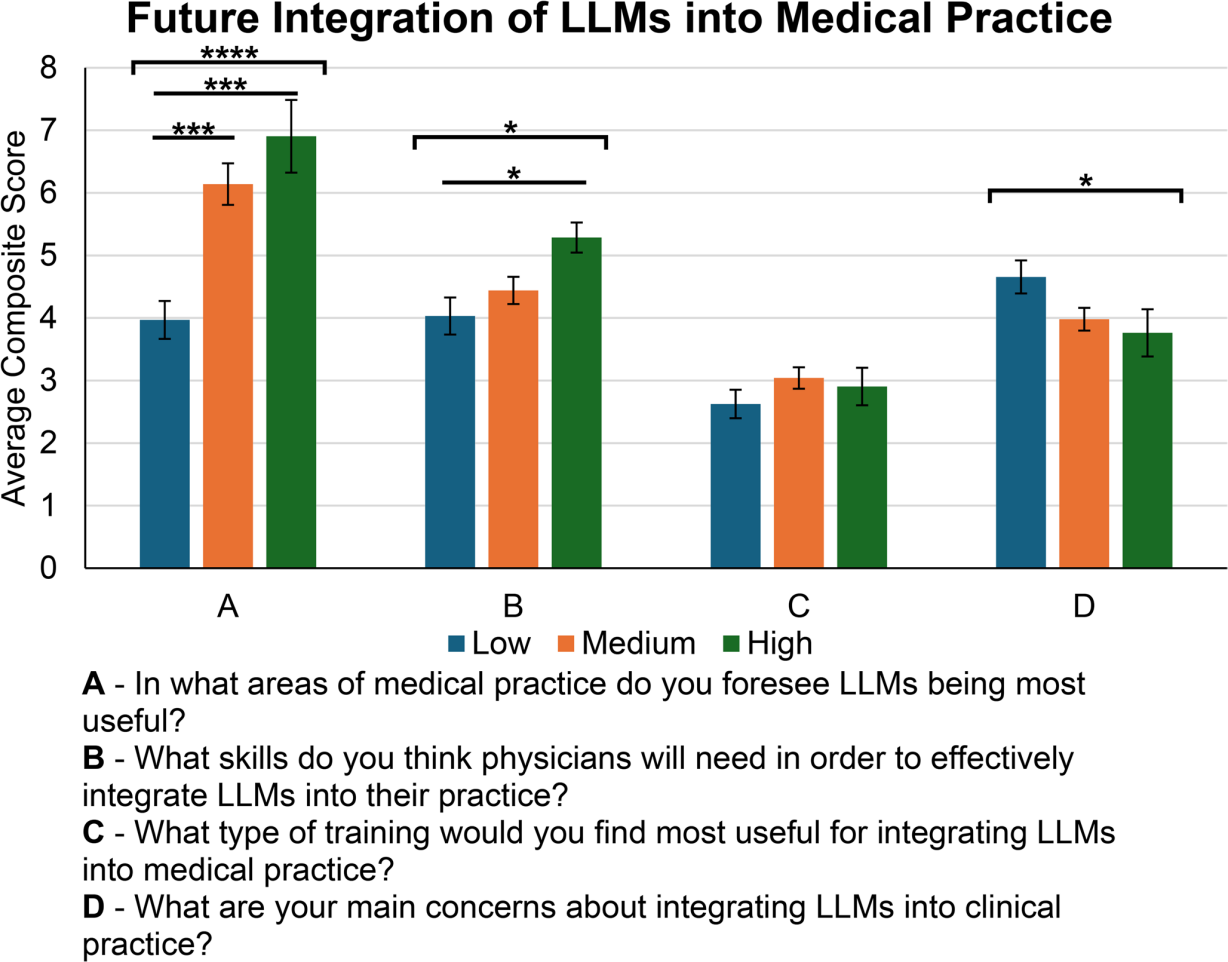
Summary of average composite scores and statistical tests for future integration of LLMs into medical practice. Kruskal-Wallis (shown with brackets) was used to test for any difference between low-, medium-, and high-LLM-use categories, with Dunn’s Post Hoc Test to test for specific comparisons (shown with line). * = *p* < 0.05; ** = *p* < 0.01; *** = *p* < 0.001; **** = *p* < 0.0001

Participants most commonly predicted LLMs would support clinical documentation and administrative tasks, and least commonly predicted being assistance with treatment management planning and continuing medical education (Table 4). Low-use participants listed fewer predicted LLM clinical applications compared to moderate- and high-use participants (Fig. 5A).

**Table 4:** Summary of responses on future integration of LLMs into medical practice

In what areas of medical practice do you foresee LLMs being most useful?	
	Participants, *n* (%)
Documenting patient interactions (e.g., medical notes)	87 (84.5%)
Assisting with administrative tasks (e.g., billing, scheduling)	81 (78.6%)
Reviewing medical literature	73 (70.9%)
Creating resources for patient education	65 (63.1%)
Clinical decision support	58 (56.3%)
Generating a differential diagnosis	55 (53.4%)
Conducting clinical research	45 (43.7%)
Patient communication (e.g., answering secure chat messages)	43 (41.7%)
Continuing medical education and professional development	41 (39.8%)
Treatment/management planning	31 (30.1%)
**What skills do you think physicians will need in order to effectively integrate LLMs into their practice?**	
	**Participants**,** n (%)**
Critical thinking to evaluate the quality and accuracy of LLM responses	99 (96.1%)
Ethical and legal understanding of LLM usage (e.g., patient confidentiality, data security)	94 (91.3%)
Technical skills to interact with LLM tools and interfaces	78 (75.7%)
Ability to incorporate LLM-generated suggestions into clinical decision-making	69 (67.6%)
Continuous learning to stay updated on advancements in LLM technology	63 (61.2%)
Collaboration skills to work alongside AI tools and other healthcare professionals	59 (57.3%)
**What type of training would you find most useful for integrating LLMs into medical practice?**	
	**Participants**,** n (%)**
Training in evaluating AI-generated recommendations	70 (68.0%)
Workshops on using LLMs for clinical decision-making	68 (66.0%)
Courses on ethics and safety in AI use for medicine	66 (64.1%)
Simulation-based learning with AI tools in clinical settings	58 (56.3%)
Online tutorials or resources for self-guided learning	35 (34.0%)
**What are your main concerns about integrating LLMs into clinical practice?**	
	**Participants**,** n (%)**
Reliability and accuracy of information provided by LLMs	93 (90.3%)
Risk of over-reliance on AI and reduced clinical judgment	88 (85.4%)
Potential for biased or incomplete recommendations	74 (71.8%)
Data privacy and security concerns	67 (65.0%)
Patient trust and understanding of AI involvement in care	55 (53.4%)
Legal and regulatory issues	50 (48.5%)
Environmental Impact	3 (2.9%)
Concern for replacement of Physicians with AI tools	1 (1.0%)

Most participants identified critical thinking as essential for integrating LLMs into clinical practice, followed by ethical and legal understanding (Table 4). Frequent LLM users identified significantly more required skills than low-users (Fig. 5B). Most participants endorsed wanting formal LLM training in: evaluating AI-generated recommendations, using LLMs for clinical decision-making, and ethics and safety in AI use for medicine (Table 4). These training recommendations did not significantly differ by LLM-usage level (Fig. 5C).

Top concerns for LLM integration into clinical practice were LLM response reliability and accuracy and over-reliance on AI and reduced clinical judgement (Table 4). There did not appear to be difference based on LLM-usage for these concerns (Fig. 5D).

## Discussion

This study examined the use of large-language model (LLM) text-generative artificial intelligence (AI) by medical students at two medical schools located within large academic institutions in different regions within the United States. In agreement with previous studies, most students reported regular LLM use, reflecting the rapid diffusion of generative AI into medical education [1]. Students noted key benefits of rapid information retrieval, medical literature summarization, and diagnostic support alongside notable limitations including inaccurate responses, shallow clinical reasoning, and privacy concerns. To our knowledge, this is the first study to relate medical students’ reported use of LLMs to outcomes such as self-rated knowledge, confidence in judging HIPAA compliance, perceived educational benefit, and expectations for clinical applications. Students with higher digital literacy reported using LLM tools more frequently (Fig. 1), yet most medical schools lack a formal education process to foster this digital literacy. We also observed wide variation in comfort and use within each institution; these differences risk creating an uneven learning environment. These findings suggest a need to integrate foundational LLM instruction into the medical curriculum not only to prepare future physicians for the clinical technologies they will likely encounter, but also to ensure a more equitable learning environment.

Students with lower LLM knowledge and less ability to distinguish between HIPAA- and non-HIPAA-compliant LLMs, which may be a proxy for digital literacy, may have lower usage rates of LLMs (Fig. 2A, B; *p* = 0.0004 and *p* = 0.016). Moreover, students with higher usage rates of LLMs appear to recognize the limitations of LLMs such as hallucination of inaccurate information (Fig. 2F, *p* = 0.024). There may be a curve in which medical students with low digital literacy do not use LLMs and thus are not subject to LLM hallucination, and users with high literacy are cognizant of LLM hallucination; however, the students with moderate digital literacy may use LLMs but without sufficient awareness of hallucinations, thus portending challenges for their education as well.

Medical education currently offers insufficient instruction in artificial intelligence and machine learning topics, leaving medical students underprepared to leverage these emerging technologies responsibly and effectively [16, 17]. In our study, medical students, regardless of their level of familiarity or experience with LLM, shared similar preferences for coursework, in concordance with findings from previous studies (Fig. 5C, *p*= 0. 394) [18–20]. Student emphasized critical thinking, ethical, and legal skills as important topics to cover, consistent with prior work identifying key ethical AI topics for instruction [21, 22]. To address these gaps, medical schools should: (1) Assess students’ digital/LLM literacy through scenario-based evaluations that test prompt design, verification strategies, source appraisal, bias detection, awareness of AI response accuracy, and proper documentation of AI use, recognizing that incoming students may vary widely in their levels of digital literacy; (2) Vet and publish an approved list of LLM tools that meet institutional standards for medical accuracy, HIPAA compliance, security, and citation transparency, with clear guidance on appropriate educational, clinical, and research use cases; (3) Develop a longitudinal AI curriculum, informed by identified gaps in the digital/LLM literacy, introduced early in medical school training, and refined over time through collaboration with computer scientists, AI researchers, medical ethicists, legal scholars, and clinicians to ensure ongoing relevance to educational, clinical, and research practices. This should be done across the clusters as all three clusters had similar rates of endorsements for such recommendations (Fig. 5C, *p* = 0.394). Core components should be identified in future studies, using medical students’ responses to this survey as one of many guides. MIT at the K-12 level (pre-university) and Harvard University at the university level do offer current AI literacy courses, and Harvard’s is based in part on a survey of its students [23, 24]. 

Limitations of the study include small sample size and response rates, although these are similar to some previous studies [25]. This may lead to selection bias which has been mitigated by offering incentives, repeat solicitation of data, and subgroup analysis. Those who tend to use AI more may differ in response rate from those who tend to use AI less, which is why subgroup analysis by AI usage was done. This study is a pilot study of just two medical schools and therefore findings cannot be generalized to all U.S. medical students; a larger study of more medical schools should be done to elicit more ideas for curricular development. These institutions may not be generalizable to all institutions, though this study addresses issues that pertain to all medical students, and the study does benefit from schools in different regions and thus geographic diversity. Additionally, the self-reported nature of data, while preserving participants’ privacy, is inferior to directly querying respondents’ LLM use digitally. This limitation may lead to self-report bias which is also mitigated by offering incentives, repeat solicitation of data, and subgroup analysis. However, the consistent responses across institutions and usage subgroups strengthens the validity of our findings.

##  Conclusion

Medical students are at the forefront of LLM usage, often adopting these tools without having institutional support and guidance as they engage in their educational and clinical activities. Medical students can offer valuable insights into how AI tools can be effectively integrated into medical education. Future areas of research would benefit from increased sample sizes, further differentiation between low-, medium-, and high-usage clusters of students, and more institutions surveyed. Furthermore, research into the cost-benefit analysis and pilot testing of this article’s recommendations would be warranted. Integrating foundational LLM instruction into the medical curriculum is essential not only to prepare future physicians for the technologies that they will encounter in clinical and research practices, but also to ensure that all medical students develop a consistent and well-informed understanding of AI-based applications and their limitations during their training.

## Electronic Supplementary Material

Below is the link to the electronic supplementary material.

Supplementary Material

## Data Availability

The datasets generated during and/or analysed during the current study are available from the corresponding author on reasonable request.

## Appendix 1

Final web-based survey instrument implemented in Google Forms (Google LLC, Mountain View, California). The survey consisted of 30 items across four sections: medical school and training stage; current use of LLMs in medical education; future integration of LLMs in medical practice; and optional demographic information. Items included multiple-choice, multi-select, and free-response formats and assessed students’ perceptions, experiences, and expectations regarding the use of generative LLMs in medical education and clinical practice.
