# Supplementary material for: A Student-Centered Approach Towards Implementing Large Language Models (LLMs) in Medical Education
Source: Med Sci Educ. 2026 Mar 21;36(3):1385–95. doi: 10.1007/s40670-026-02675-x (PMC13356141; doi:10.1007/s40670-026-02675-x)
Supplement: Supplementary file 1 — Supplementary Material [file 40670_2026_2675_MOESM1_ESM.docx]

**Survey regarding Medical Student Use of Large Language Models (LLMs)**

We want to hear **how medical students like you** are using AI-powered large language models (LLMs) like ChatGPT in your studies. Most research on this topic comes from academic educators, which may not fully capture how medical students actually use these tools. Your input will help bridge this gap and guide future developments.

This survey takes just a few minutes and is **completely** **anonymous**. The results will be analyzed in aggregate and will not be individually identifiable. At the end, you'll have the option to enter a **raffle for a $25 Amazon gift card** by submitting your email separately.

Our study has been determined to be IRB exempt at the University of Michigan (HUM00267318) and the University of Pennsylvania (858189).

Your perspective matters—help us understand AI use in medical education!

**Q1** - Which medical school are you currently enrolled in?*

- University of Michigan
- University of Pennsylvania

**Q2** - Which year of medical school are you in?*

- M1
- M2 (pre-clerkship)
- M2 (clerkship)
- M3 (clerkship)
- M3 (post-clerkship)
- M4
- MSTP - 1st year of PhD
- MSTP - 2nd year of PhD
- MSTP - 3rd year of PhD
- MSTP - 4th year of PhD
- Leave of Absence or Gap Year
- Other:

**Current Use of LLMs in Medical Education**

**Q3** - Which of the following generative large language models (LLMs) have you used within a medical school context (ie. educational, clinical, research or administrative purposes within your medical school experience), and how often?*

|  | Never | A few times per year | Once per month | A few times per month | Once per week | A few times per we | Once per day | Multiple times per day |
| --- | --- | --- | --- | --- | --- | --- | --- | --- |
| Anthropic Claude |  |  |  |  |  |  |  |  |
| DeepSeek |  |  |  |  |  |  |  |  |
| Google Gemini |  |  |  |  |  |  |  |  |
| Meta Llama |  |  |  |  |  |  |  |  |
| Microsoft Copilot |  |  |  |  |  |  |  |  |
| OpenAI ChatGPT |  |  |  |  |  |  |  |  |
| Perplexity |  |  |  |  |  |  |  |  |

**Q4** - If you use LLM(s) other than those listed above, please add those here and describe how often you use them using the same categories displayed above.

Your answer

**Q5** - Do you currently pay for access to any generative LLMs, such as ChatGPT Pro?*

- No
- Yes

**Q6** - How would you rate your knowledge of LLMs with regards to the following?*

|  | Poor | Below Average | Average | Above Average | Excellent |
| --- | --- | --- | --- | --- | --- |
| Capabilities (understanding what LLMs can do) |  |  |  |  |  |
| Applications (understanding which tasks can be assisted by LLMs) |  |  |  |  |  |
| Prompt-engineering (understanding how to input to get desired outputs) |  |  |  |  |  |
| Limitations/Challenges (understanding what LLMs can't do) |  |  |  |  |  |

**Q7** - In which of the following medical school contexts have you used LLMs, and how helpful have they been?*

|  | Didn't use it | Used it but didn't help | Used it and was helpful |
| --- | --- | --- | --- |
| Generating a differential diagnosis |  |  |  |
| Generating interactive clinical vignettes |  |  |  |
| Summarizing clinical vignettes |  |  |  |
| Generating explanations to review multiple-choice questions |  |  |  |
| Generating practice questions |  |  |  |
| Generating flash cards |  |  |  |
| Summarizing medical literature |  |  |  |
| Summarizing patient information |  |  |  |
| Looking up fact-based questions |  |  |  |
| Assisting with research data analysis |  |  |  |
| Assisting with writing research abstract/manuscript |  |  |  |

**Q8** - Have you ever used HIPAA-compliant LLMs to analyze patient cases?*

- No
- Yes

**Q9** - How confident are you at distinguishing between HIPAA-compliant and non-HIPAA-compliant LLMs?*

- Not at all confident – I don't know what HIPAA compliance means for LLMs.
- Not confident – I’m unsure about the criteria for HIPAA compliance in LLMs.
- Somewhat confident – I have a general understanding but may need guidance to confirm compliance.
- Very confident – I can easily identify whether an LLM meets HIPAA compliance standards.

**Q10** - In your current phase of training (pre-clinical, clerkship, post-clerkship), how frequently have instructors, faculty, or educators at your medical school/hospital asked you to use LLMs?*

- Never
- A few times per year
- Once per month
- A few times per month
- Once per week
- A few times per week
- Once per day
- Multiple times per day

**Q11** - What features do you find most helpful when using LLMs for medical education? (Select all that apply)*

- Speed of information retrieval
- Clarity of explanations
- Ability to generate detailed responses to complex questions
- Availability of interactive, case-based learning
- Personalized or tailored responses to specific queries
- Help with study organization or planning
- Other:

**Q12** - What challenges or limitations have you encountered when using LLMs for your medical education? (Select all that apply) *

- Inaccurate (factually incorrect) information
- Outdated (not current) information
- Lack of depth in clinical reasoning
- Difficulty understanding complex medical jargon
- Lack of clinical context in responses
- Ethical concerns (e.g., misinformation, privacy)
- Limited ability to answer highly specialized questions
- Other:

**Q13** - If you answered inaccurate or outdated information in the previous question, please describe how you discovered the error.

Your answer

**Q14** - To what extent do you agree with the following statement? "The use of Large Language Models (LLMs) has significantly improved my medical education by enhancing my understanding of medical concepts, providing useful resources, and assisting with learning tasks."*

- Strongly disagree
- Somewhat disagree
- Neutral
- Somewhat agree
- Strongly agree

**Future Integration of LLMs in Medical Practice**

**Q15** - In what ways do you think LLMs will shape medical education in the future?*

Your answer

**Q16** - How do you foresee LLMs impacting clinical practice?*

Your answer

**Q17** - In what areas of medical practice do you foresee LLMs being most useful? (Select all that apply)*

- Assisting with administrative tasks (e.g., billing, scheduling)
- Clinical decision support
- Conducting clinical research
- Continuing medical education and professional development
- Creating resources for patient education
- Documenting patient interactions (e.g., medical notes)
- Generating a differential diagnosis
- Patient communication (eg., answering secure chat messages)
- Reviewing medical literature
- Treatment/management planning
- Other:

**Q18** - What skills do you think physicians will need in order to effectively integrate LLMs into their practice? (Select all that apply) *

- Critical thinking to evaluate the quality and accuracy of LLM responses
- Technical skills to interact with LLM tools and interfaces
- Ethical and legal understanding of LLM usage (e.g., patient confidentiality, data security)
- Ability to incorporate LLM-generated suggestions into clinical decision-making
- Collaboration skills to work alongside AI tools and other healthcare professionals
- Continuous learning to stay updated on advancements in LLM technology
- Other:

**Q19** - Should medical education include specific training on how to use LLMs in clinical practice?*

- No
- Yes

**Q20** - Please elaborate on the previous question*

Your answer

**Q21** - What type of training would you find most useful for integrating LLMs into medical practice? (Select all that apply)*

- Workshops on using LLMs for clinical decision-making
- Courses on ethics and safety in AI use for medicine
- Training in evaluating AI-generated recommendations
- Simulation-based learning with AI tools in clinical settings
- Online tutorials or resources for self-guided learning
- Other:

**Q22** - What are your main concerns about integrating LLMs into clinical practice? (Select all that apply)*

- Reliability and accuracy of information provided by LLMs
- Risk of over-reliance on AI and reduced clinical judgment
- Potential for biased or incomplete recommendations
- Patient trust and understanding of AI involvement in care
- Data privacy and security concerns
- Legal and regulatory issues
- Other:

**Q23** - Please use this free text space to add any comments about other useful ways you use LLMs, other resources that you think would be helpful, anticipations or concerns about LLM use in medical education, or anything else you'd like to share.

Your answer

**Demographics**

**ALL of the following questions are OPTIONAL. Demographic questions will be used to analyze our results in aggregate and will help identify patterns in responses.**

**Q24** - What is your age?

Your answer

**Q25** - How is your gender identity best characterized?

- Man
- Woman
- Other:

**Q26** - How is your sex at birth best characterized?

- Male
- Female
- Other:

**Q27** - Which of the following best characterizes your race?

- American Indian or Alaska Native
- Asian
- Black or African American
- Native Hawaiian or Other Pacific Islander
- White
- Other:

**Q28** - Please feel free to further specify your race, if desired:

Your answer

**Q29** - Which of the following best characterizes your ethnicity?

- Hispanic or Latino
- Not Hispanic or Latino

**Q30** - Please feel free to further specify your ethnicity, if desired:

Your answer
